# Identification of novel 7-hydroxycoumarin derivatives as ELOC binders with potential to modulate CRL2 complex formation

**DOI:** 10.1038/s41598-025-88166-2

**Published:** 2025-01-29

**Authors:** Yonghyeok Kim, Seon Jeong Baek, Eun-Kyung Yoon, Minhee Choi, Jung-Hoon Kim, Kyungtae Kim, Chi Hoon Park, Byung Il Lee

**Affiliations:** 1 Research Institute, National Cancer Center, Goyang-si, 10408 Gyeonggi Republic of Korea; 2 Department of Cancer Biomedical Science, National Cancer Center Graduate School of Cancer Science and Policy, Goyang-si, 10408 Gyeonggi Republic of Korea; 3 Department of Medicinal Chemistry and Pharmacology, University of Science and Technology, Daejeon, 34113 Republic of Korea; 4 Therapeutics & Biotechnology Division, Korea Research Institute of Chemical Technology, Daejeon, 34114 Republic of Korea

**Keywords:** 7-hydroxycoumarin, Umbelliferone, E3 ligases, ELOC, CRL2, CRL5, Ubiquitin ligases, X-ray crystallography

## Abstract

The VHL-containing cullin-RING E3 ubiquitin ligase (CRL2^VHL^) complex is an E3 ligase commonly used in targeted protein degradation (TPD). Hydroxyproline-based ligands that mimic VHL substrates have been developed as anchor molecules for proteolysis-targeting chimeras (PROTACs) in TPD. To expand the chemical space for VHL ligands, we conducted fragment screening using VHL–ELOB–ELOC (VBC) proteins. We found that certain 7-hydroxycoumarin derivatives (7HCs), rather than VHL, would bind to the ELOC component of the VBC complex. The 7HC binding site overlapped with the CUL2 binding interface on ELOC but did not overlap with the CUL5 binding interface, suggesting that 7HCs may influence the formation of CRL2 but not CRL5. Although the binding affinities of these 7HCs to the VBC complex were relatively low, they represent novel and promising foundational agents for the development of chemical probes or inhibitors that target ELOC-containing CRLs.

**Supplementary Information:**

The online version contains supplementary material available at 10.1038/s41598-025-88166-2.

## Introduction

In eukaryotic cells, ubiquitination is a major form of post-translational protein modifications in which ubiquitin, an 8.6-kDa regulatory protein, covalently conjugates to the amine group of the lysine residue or N-termini of substrate proteins. Ubiquitination plays a key role in the regulation of various biological processes, including protein degradation and localization^1–3^. Ubiquitination is a multistep enzymatic process that involves ubiquitin-activating enzyme (E1), ubiquitin-conjugating enzyme (E2), and ubiquitin ligase (E3). To date, two E1 enzymes, approximately 40 E2 enzymes, and more than 600 E3 enzymes have been identified^4^. E3 ubiquitin ligases are a large and diverse family of proteins that are categorized into three classes: Really Interesting New Gene (RING), Homologous to the E6-AP Carboxyl Terminus (HECT), and RING between RING (RBR). The RING family of E3 ubiquitin ligases comprises the largest set of E3 ubiquitin ligases, and includes approximately 600 that are further classified as monomeric, homodimeric, heterodimeric, and multisubunit RING E3 ubiquitin ligases^5,6^.

Targeted protein degradation (TPD) technologies, which exploit endogenous protein degradation machinery to degrade proteins of interest (POIs), have emerged as a novel therapeutic approach to modulating disease-associated proteins^5^. While traditional small-molecule inhibitors diminish protein functioning, TPD technologies inhibit the functions of the specific protein through the degradation of target proteins^7^. One of the major TPD technologies is the proteolysis-targeting chimeras (PROTACs). PROTACs utilize the ubiquitin–proteasome system (UPS) for protein degradation. PROTACs are heterobifunctional molecules consisting of two ligand components: an “anchor” that binds to an E3 ubiquitin ligase and a “warhead” that binds to a POI^8^. The two components are connected using a chemical linker. By inducing the proximity of an E3 ubiquitin ligase and the POI using PROTAC, ubiquitin can be conjugated to the recruited POIs via E3 ubiquitin ligase enzymatic activity^9^. The resulting polyubiquitin-conjugated POIs are recognized and degraded by proteasomes into small peptides. PROTAC technology has the potential to target “undruggable” proteins, enhance therapeutic effects at low doses, and reduce dosing frequency with fewer side effects^10^. Moreover, PROTACs can be recycled for subsequent rounds of degradation^11^.

Two cullin-RING family E3 ubiquitin ligases (CRLs), CRL2^VHL^ and CRL4^CRBN^, are commonly used in TPD technology^5^. These CRLs are members of the multisubunit RING ubiquitin E3 ligases with a cereblon (CRBN, substrate receptor subunit)–DNA damage-binding protein 1 (DDB1, adaptor subunit) and von Hippel–Lindau tumor suppressor (VHL, substrate receptor subunit)–Elongin B (ELOB, adaptor subunit)–Elongin C (ELOC, adaptor subunit) (VBC) as the substrate receptor–adaptor components. Immunomodulatory drugs (IMiDs), such as thalidomide, pomalidomide, and lenalidomide, are representative CRBN ligands widely used as anchor molecules for PROTACs^5^. Hydroxyproline (HyPro or HyP)-based ligands are used as anchor molecules targeting VHL^5,12–15^. As anchor molecules are critical factors in PROTAC technology, extensive efforts have been made to identify new anchor molecules. VHL has been regarded as an emerging molecular target for PROTAC anchor discovery, and numerous small-molecule ligands targeting VHL for the discovery of VHL inhibitors or PROTAC anchor molecules have been discovered^16^.

Therefore, in this study, we originally aimed to identify PROTAC anchor molecules targeting VHL through fragment library screening. However, we serendipitously discovered novel molecules that could bind to ELOC. Our molecules bound to the previously reported “ELOC site”, to which some small molecules and cullin-2 (CUL2)-derived peptides can bind^17,18^. Our compounds validated the druggability of previously reported small-molecule binding sites in ELOC again and can be a starting point for development of ELOC site-targeting ligands.

## Results and discussion

### High-throughput screening for the identification of new VBC binders using fragment chemical library

We conducted chemical library screening to develop VHL binders with new chemical scaffolds. First, screening of the recombinant VBC protein with a Maybridge fragment library set (approximately 1,500 chemicals, 300 µM final concentration) by an FP assay resulted in 15 hit compounds that exhibited a decreased fluorescence polarization signal (10% FP signal decrease cut-off compared with the negative control). Next, the 15 hit chemicals were further tested by a thermal-shift assay and narrowed down to three hit chemicals that exhibited a T_m_ shift of more than 0.5 ℃. One of the hit compounds from these screening experiments was a coumarin derivative (7HC; 7HC_25(org) in Supplementary Table S1), and further structural studies were performed with coumarin derivative compounds.

### Crystal structures of VBC in complex with the 7-hydroxycoumarin derivative

**Fig. 1:**
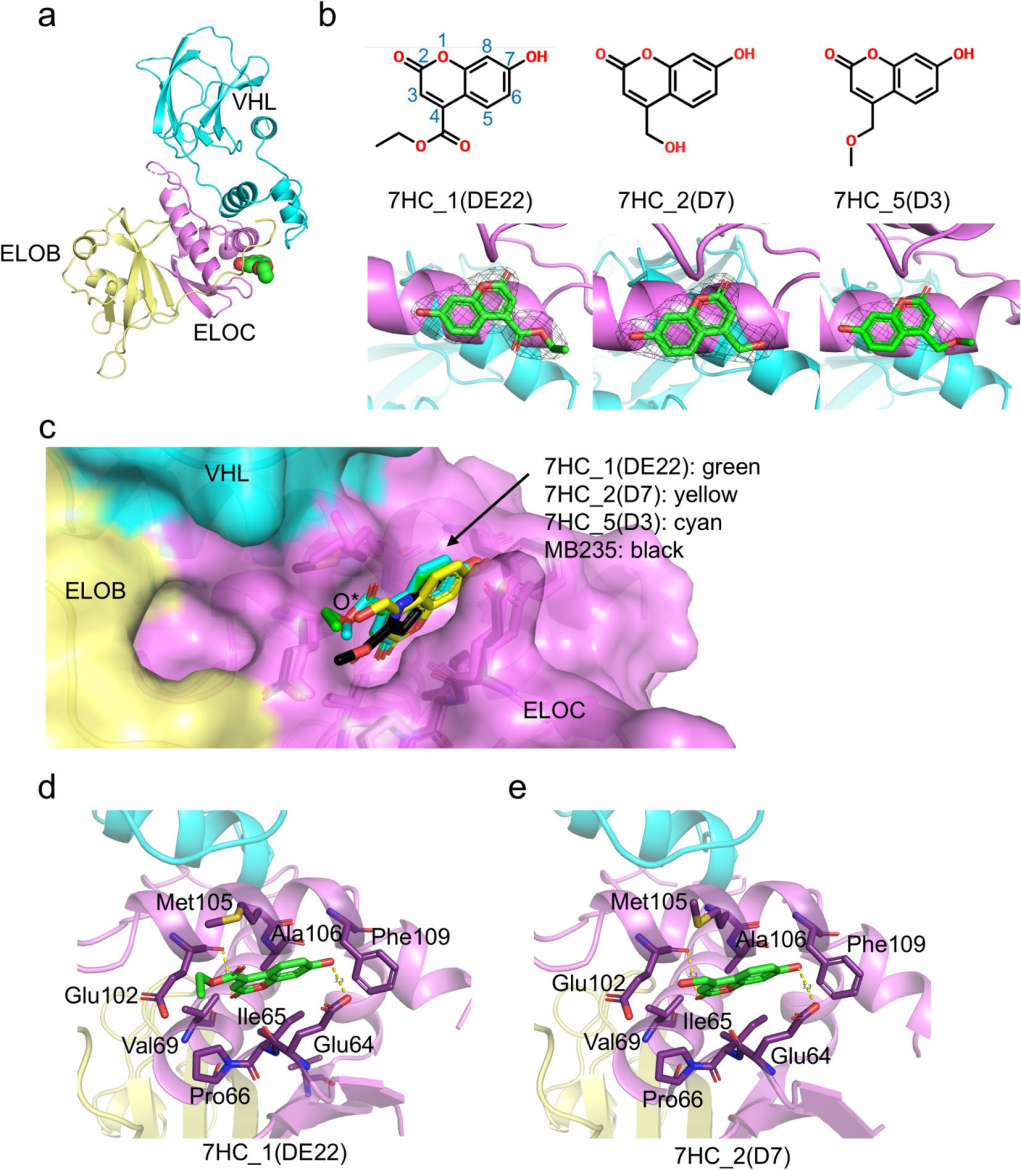
Overall structures of VBC in complex with fragment ligands. (**a**) Overall structures of the VBC−7HC_1(DE22) complex. Each protein component is colored in cyan (VHL), pale yellow (ELOB), and violet (ELOC). The ligand is shown in the sphere model. (**b**) Chemical structures and 2mFo-DFc OMIT maps for 7HC_1(DE22), 7HC_2(D7), and 7HC_5(D3). The numbering for the 7HC ring is also shown. The map contour levels were 1.5 σ. (**c**) Superimposition with ligand-bound VBC structures. 7HC_1(DE22) is drawn in green, 7HC_2(D7) in yellow, 7HC_5(D3) in cyan, and MB235 in black (PDB code: 6GMN)^17^. O*: position of the O3 atom in the 7HC_1(DE22) and 7HC_1(DE22) molecules and O4 atom in the 7HC_5(D3) molecule. (**d**) Detailed views of interactions between VBC and 7HC_1(DE22). (**e**) Detailed views of interactions between VBC and 7HC_2(D7).

In the early stages of the crystallographic study with three hit compounds after the thermal-shift assay (Supplementary Fig. S1), electron density that appeared to belong to the 7HC_25(org) molecule was observed at the ligand-binding site of VHL. Based on this, further studies using 7HC derivatives were conducted. To determine the crystal structures of VBC in complexes with coumarin derivative compounds, we attempted co-crystallization and soaking with each of the 25 compounds (2–5 mM of 25 purchasable coumarin derivatives, as listed in Supplementary Table S1). Successful results were obtained using soaking for 7HC_1(DE22) and 7HC_5(D3), and co-crystallization for 7HC_2(D7). Three 7HC derivative-bound VBC structures were determined and refined at resolutions of 2.60, 2.46, and 3.00 Å (Supplementary Table S2). The VBC complex exhibited heterotrimeric structures of VHL, ELOB, and ELOC, as described previously (Fig. 1a)^19^. VHL has two β-sheet rich domains (β-domain) and α-helical domains (α-domain), and it is held by two linkers and a polar interface. The C-terminal α-helix of VHL folded back to the β-domain, stabilizing the overall fold. The ELOC exhibited an α/β roll-fold. The C-terminal α-helix of ELOC and its long preceding loop interacted with the large VHL α-domain surface and a small part of the β-domain. ELOB showed a ubiquitin-like α/β roll fold and bound to ELOC in the opposite region of the VHL-binding site in ELOC. However, the long C-terminal end loop of ELOB covered one side of the ELOC and extended to the α-domain of VHL (Fig. 1a). The ELOB–ELOC binding interface formed by two juxtaposed β-sheets generates an intermolecular sheet^19^. Similar to the previously reported VBC structures, four VBC complex molecules existed in the asymmetric unit of the crystal. Clear electron density maps for 7HCs (7HC_1(DE22), 7HC_2(D7), and 7HC_5(D3)) could be observed (Fig. 1b). The 7HC_1(DE22) molecule was modeled in three chains (B, H, and K), VBC−7HC_2(D7) was modeled in all four chains (B, E, H, and K), and 7HC_5(D3) was modeled in only one chain (the K chain). However, these electron density maps were not for the VHL molecule but for the ELOC protein (Fig. 1). We concluded later that the observed electron density in the VHL did not originate from the 7HC_25(org). The slight decrease in the FP signal during the biochemical screening may have partly been due to the autofluorescence of the 7HC compound. The three 7HCs bound to ELOC in an identical manner, and hydrophobic interactions were dominant (Fig. 1c-e). When analyzed with two higher-resolution structures (7HC_1(DE22) and 7HC_2(D7)-bound VBC complexes, PDB codes of 8ZVJ and 8ZV8, respectively), eight residues (Glu64, Ile65, Pro66, Val69, Glu102, Met105, Ala106, and Phe109) in ELOC were close to 7HC_1(DE22) and (7HC_2(D7) ligands within 4 Å distance (Fig. 1d, e). When we analyzed protein–ligand interactions with the PLIP server (https://plip-tool.biotec.tu-dresden.de/plip-web/plip/index; we excluded interactions with neighboring VBC molecules due to crystal packing)^20^, the core benzopyran moiety of 7HCs formed hydrophobic interactions with the aliphatic side chains of Glu64, Ile65, Glu102, and Met105. Met105 participated in hydrophobic interactions in all seven ELOC structures of 7HC_1(DE22) and 7HC_2(D7) (chains B, H, and K in 7HC_1(DE22); chains B, E, H, and K in 7HC_2(D7)). Hydrophobic interactions between the benzopyran moiety and Glu102 were missing in one ELOB structure (chain B of 7HC_2(D7)) but were retained in the other six ELOC structures. Hydrophobic interactions between the benzopyran moiety and Glu64 were observed in chains B and E in 7HC_2(D7). Ile65−benzopyran interactions were found in chain-H structures of 7HC_1(DE22) and 7HC_2(D7). Interaction analysis using the PyMOL program (find the polar contacts’ option) suggested additional polar interactions (Fig. 1d, e, yellow lines). The 2-oxo group of each 7HC derivative formed a polar interaction with the main-chain carbonyl group of Glu102, and the 7-hydroxy group also interacted with the side chain of Glu64.

As mentioned above, two ELOC-binding ligands, MB235 and MB1200, have also been described previously^17^. When we superimposed our three 7HC structures with the VBC−MB235 complex structures, the ligand-binding sites were identical (Fig. 1c).

A structural comparison of ligand-free and ligand-bound ELOC showed that the hydrophobic pocket structure was nearly identical (Supplementary Fig. S2). These results show that ligand recognition is likely due to conformational selection, rather than an induced fit^21^.

### Biochemical validation of VBC-ligand interaction

We selected some compounds and tested their binding affinities using SPR (Supplementary Table S1). Five coumarin derivatives (7HC_6(D4), 7HC_7(D5), C_9(D8), 7HC_21(D20), and 7HC_23(D22)) did not interact with VBC at all, and two 7HCs, 7HC_1(DE22) and 7HC_2(D7), whose protein–ligand complex structures were successfully determined, showed weak binding to VBC (Supplementary Fig. S3). However, we could not determine the binding affinities (K_D_) of these 7HCs because SPR responses did not reach saturation and conducting an experiment with high ligand concentration was impossible due to the poor solubility of ligands.

**Fig. 2:**
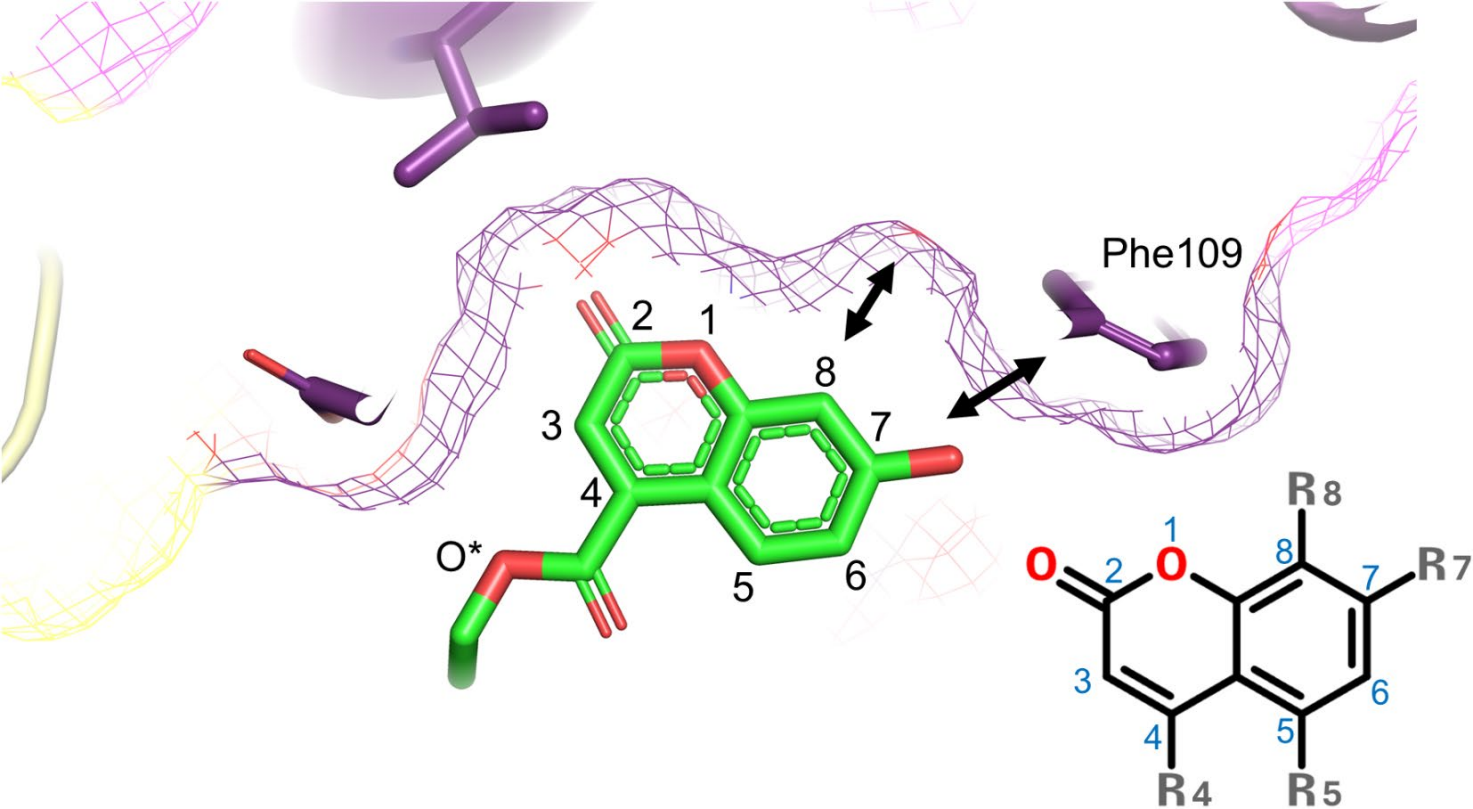
Sliced view of 7HC derivative binding pocket surface showing the structural basis of no binding activities by C7- and C8-position chemical modifications (R7 and R8). The 7HC_1(DE22) structure is shown as the representative. O*: position of the O3 atom in the 7HC_1(DE22).

According to our SPR results and structural studies, the substitution of the 7-hydroxyl group with other chemical functional groups (R7; C_4(D2), C_9(D8), C10_(D9), C12(D11), C_13(D12), C_14(D13), C_15(D14), C_16(D15), C_17(D16), C_18(D17), and C_19(D18)) showed negative effects for VBC−ligand interaction, mainly because of steric clash by overlapping with Phe109 (Fig. 2). Coumarin derivatives with chemical groups at the C8 position (R8; C_11(D10), C_19(D18), 7HC_20(D19), 7HC_21(D20), 7HC_22(D21), 7HC_23(D22), and 7HC_24(D23)) did not bind to ELOC because of steric clashes (Fig. 2). Three 7HCs (7HC_1(DE22), 7HC_2(D7), and 7HC_5(D3)) had functional groups at the C4 position (R4) and VBC-binding activity. Owing to the lack of in vitro VBC-binding activity for the other C4 position derivatives (7HC_3(D1), 7HC_6(D4), and 7HC_7(D6)), we could not determine the structural basis. We presumed that the oxygen atoms in the R4 group (O3 atom in the PDB code of 8ZVJ and 8ZV8, and O4 atom in the PDB code of 9IPW; denoted as O* in Figs. 1c and 2) may have contributed to ELOC binding. Although no molecule was included in the refined model, very weak electron density corresponding to a water molecule was observed between the O* atom and the side chain of Glu102 in certain ELOC molecules within the asymmetric unit, suggesting a water-mediated interaction.

### Structural basis of the regulation of ELOC-cullin interactions by 7HC derivatives

**Fig. 3:**
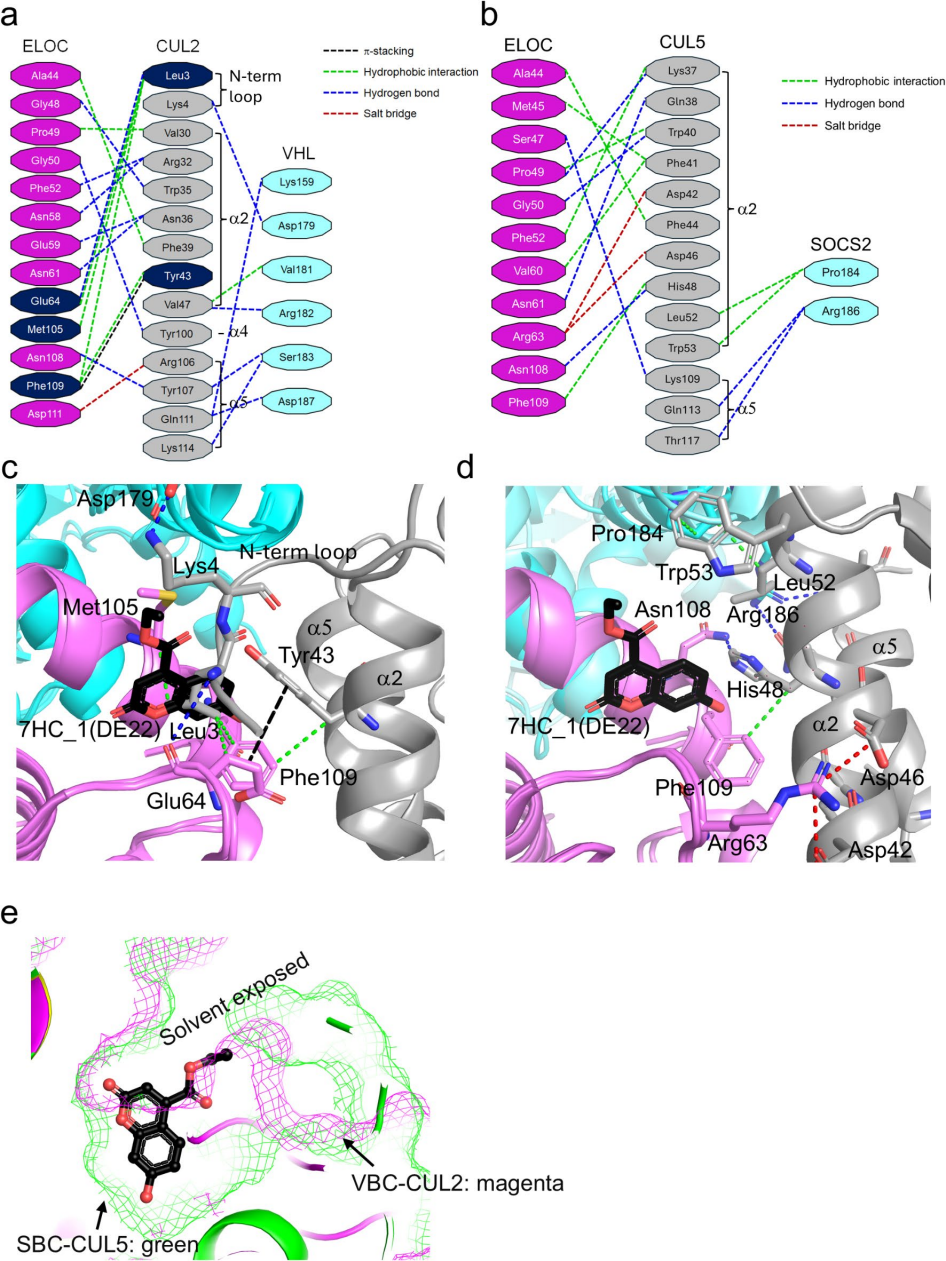
Structural analyses of 7HCs in VBC−CUL2 and SBC−CUL5 interactions. (**a**) Diagram representing VBC−CUL2 interaction. Residues interfered with by 7HCs are colored in navy. (**b**) Diagram representing SBC−CUL5 interaction. (**c**) Structural superposition of VBC−7HC_1(DE22) complex structure with VBC−CUL2 structure. (**d**) Structural superposition of VBC−7HC_1(DE22) complex structure with SBC−CUL5 structure. PPIs were analyzed with the PLIP server. VHL or SOCS2 were colored in cyan, ELOC in violet, and cullin proteins in grey. The 7HC_1(DE22) ligand is colored in black. Interactions representing the PPIs around the ligand are indicated in blue (hydrogen bond), green (hydrophobic interaction), red (salt bridge), and black (π-stacking) lines, respectively. The secondary structures of each cullin structure are marked as N-term loop, α2, and α5, respectively. (**e**) Structural comparison of 7HCs binding pockets in VBC−CUL2 (magenta) and SBC−CUL5 (green) by sliced view surface representation of protein structures.

During the early stages of studies on Elongin, ELOB and ELOC formed elongated complexes with ELOA. It is a positive regulator of RNA polymerase II and suppresses transient pausing of the enzyme^22–25^. In addition, ELOBC is an adaptor subunit of CRL2/5 E3 ligases, bridging a substrate receptor subunit (e.g., VHL or SOCS proteins) and cullin proteins (CUL2 or CUL5)^26^. The binding selectivity of CUL2 or CUL5 is usually determined by the amino acid sequence and three-dimensional structure of the substrate receptor in CRLs. The VHL-box in VHL contains two helices, BC- and CUL2-box, showing selectivity for CUL2, and SOCS proteins have a SOCS-box that comprises BC- and CUL5-box interacting with CUL5^27,28^. Structural snapshots of VBC−CUL2 and SOCS2−ELOB−ELOC(SBC)−CUL5 interactions have been reported (PDB codes 4WQO, 5N4W, and 4JGH)^29–31^. Although the structure of the VBC−CUL2 complex shares an ELOBC with that of the SBC−CUL5 complex, the binding mode of the VBC−CUL2 interaction is not identical to that of SBC−CUL5 (Supplementary Fig. S4). Compared with CUL5 in the complex structure, the α2 helix of the CUL2 molecule was slightly pushed outward from the ELOC, forming fewer interactions than SBC−CUL5 (α2). Instead, the N-terminal loop region of CUL2 was inserted into the hydrophobic pocket of ELOC, and Leu3 and Lys4 in the loop and residues in the α4 and α5 helices participated in protein–protein interactions (PPIs), generating a greater number of total interactions in VBC−CUL2 (Supplementary Fig. S4; Fig. 3a, b). The N-terminal region of CUL5 moved outward and did not participate in the SBC−CUL5 interaction. Instead, the second helix of CUL5 (α2) moved closer to the ELOC, resulting in additional interactions compared with CUL2.

We further analyzed whether 7HCs have the potential to interfere with PPIs between VBC (or SBC) and cullin by superimposing our VBC−7HCs complex structures on previously reported VBC−CUL2 and SBC−CUL5 structures^29,30^. Structural superposition revealed that the 7HCs overlapped mainly with Leu3 of CUL2 and possibly interfered with the interaction between VBC and CUL2 (Fig. 3c). Specifically, they blocked a hydrogen bond between Leu3 (CUL2) and Glu64 (ELOC) and several hydrophobic interactions between Leu3 of CUL2 and Glu64, Met105, and Phe109 of ELOC. 7HCs can also indirectly interfere with the hydrophobic and π-stacking interactions between Phe109 (ELOC) and Tyr43 (CUL2). In a previous study, a mutation in Leu3 in CUL2 resulted in significantly decreased VBC interactions^29,31^. Therefore, we could hypothesize that our 7HCs interfere with VBC−CUL2 interactions. According to the SBC−CUL5 complex structure, 7HCs are expected not to inhibit SBC−CUL5 interactions because the N-terminal region of CUL5 does not participate in interactions, and the tyrosine residue (Tyr43) in CUL2 (which makes hydrophobic and π-stacking interactions with Phe109 of ELOC) is replaced with histidine (His48) in CUL5 (Fig. 3b, d). Although His48 still forms hydrophobic interactions with Phe109, the 7HCs may not block this interaction (Fig. 3d). The surface representations of the VBC−CUL2 and SBC−CUL5 structures show that the 7HCs binding site was disrupted by the VBC−CUL2 interaction but retained the SBC−CUL5 structure (Fig. 3e, Supplementary Fig. S5). Although it is difficult to conclude that 7HC can modulate the formation of CRL2 or CRL5 due to the low binding affinity of 7HC ligands, we hypothesize that certain 7HC derivatives may have the potential to modulate CRL2 or CRL5.

As previously mentioned, ELOBC binds to the RNA polymerase II complex via an ELOA anchor molecule. The cryoelectron microscopy (cryo-EM) structure of the RNA polymerase II−Elongin complex (PDB code: 8OF0) has been recently described^25^. The 7HC binding site in ELOC is not masked by other proteins and may not interfere with the RNA polymerase II−Elongin interaction (Supplementary Fig. S6).

## Conclusion

In this study, we determined three crystal structures of VBC complexed with three 7HCs; 7HC is a natural product that absorbs ultraviolet radiation and has several alternative names, including umbelliferone, hydrangine, and skimmetine^32^. 7HC and its derivatives exert numerous pharmacological activities against diabetes, cardiovascular diseases, neurodegenerative diseases, inflammatory disorders, various cancer types, and microbial infections^32^. These pharmacological activities are attributed to the broad protein-binding properties of 7HCs. We found many 7HC-bound crystal structures, including γ-chymotrypsin, β-ketoacyl synthase, and sulfotransferase, in the Protein Data Bank, suggesting broad protein-binding activity of 7HC^33–35^. Here, we present direct evidence that a subset of 7HCs binds to ELOC. As 7HCs are not strong ELOC binders, our 7HCs could be a possible starting point for the development of chemical probes or inhibitors for ELOBC-containing CRLs.

A previous peptide-based study showed effective blockage of the VBC−CUL2 interaction targeting our 7HC derivative binding site, suggesting a possible inhibitor with a 7HC-based scaffold^18^. Inhibitors targeting CRLs have been developed and tested in clinical studies on patients with cancer^26,36^. Neddylation inhibitors, which indirectly inhibit the activity of CRLs and PPI inhibitors that block the E3 ligase complex association, have been developed^36,37^. Although our 7HC hits are not currently effective inhibitors, owing to their relatively low binding affinities, they are still applicable for the development of CRL association inhibitors. These 7HCs are small fragment-like compounds that can be expanded into larger and more efficient lead compounds.

7HCs are fluorescent molecules that absorb ultraviolet radiation^38^. As our crystal structure suggested the direct binding of 7HCs without CRL5 formation interference, our 7HCs may also be used for the development of CRL5 chemical probes with fluorescent signals.

From another point of view, if 7HCs are designed not to affect CRL5 formation, potential anchor molecules for PROTACs that connect ELOC and POIs should be considered. However, an investigation into the structure of the E3 ligase holoenzyme^39^ revealed that ELOC is too far from substrates and is blocked by substrate receptor components, such as VHL and SOCS proteins, from reaching POIs (Supplementary Fig. S7). Although PROTACs with ELOC anchors have the advantage of utilizing numerous E3 ubiquitin ligases, rather than a single E3 ubiquitin ligase, the feasibility of these PROTACs is questionable.

## Methods

### Purification of VBC complex

The VHL gene (residue 54−213) was amplified by polymerase chain reaction (PCR) and cloned into the BamHI/EcoRI sites of the pHis vector (modified pET28b vector). A hexahistidine (6×His) tag was inserted into the N-terminus of VHL for high-purity protein purification. The ELOB (residue 1−104) and ELOC (residue 17−112) genes were cloned into the pACYCDuet-1 vector. pHis-VHL_54−213_ and pACYCDuet-ELOB_1−104_-ELOC_17−112_ were co-expressed in BL21(DE3) cells that were cultured in Terrific Broth at 37 ℃. Upon the cells reaching an OD_600_ of 0.7, 0.5 mM isopropyl β-D-thiogalactoside (IPTG) was added to the cell culture, and the cells were further cultured at 18 ℃ for 21 h. Subsequently, the cells were harvested by centrifugation at 4 °C and 11,325 ×g for 10 min. The cell pellet was resuspended in lysis buffer (20 mM Tris-HCl pH 8.0, 500 mM NaCl, and 10 mM imidazole) and passed twice through a microfluidizer (PICOMAX, Micronox, Sungnam, Korea) at 1,000 bars. After centrifugation at 48,400 ×g for 1 h, the supernatant was collected and incubated in Ni-NTA resin (Cytiva, Marlborough, MA, USA) with gentle stirring for 1 h under ice-cold conditions. After extensive washing, proteins were eluted from the Ni-NTA resin using an elution buffer (20 mM Tris-HCl pH 8.0, 500 mM NaCl, and 500 mM imidazole). The eluted protein was incubated with tobacco etch virus (TEV) protease for 3 days at 4 ℃ to remove the His tag. The cleaved VBC protein was collected in the flow-through fraction after passing through the Ni-NTA resin open column. The VBC complex-containing flow-through was loaded onto a HiTrap Q HP anion-exchange column (Cytiva, Marlborough, MA, USA) equilibrated with IEX buffer (20 mM bis-Tris (pH 7.0) and 1 mM dithiothreitol (DTT)). VBC was eluted using a linear gradient of 1 M NaCl in the IEX buffer. The protein was further purified using a HiLoad 16/600 Superdex 75 prep grade column (Cytiva, Marlborough, MA, USA) with final storage buffer (20 mM Bis-Tris, pH 7.0, 150 mM NaCl, and 1 mM DTT). Protein was concentrated to 8–20 mg/mL using Amicon Centrifugal Filter Units (Merck Millipore, Burlington, MA, USA) and stored at − 80 ℃ for biochemical assays and crystallization.

### Fragment screening

A fluorescence polarization (FP) assay was used to identify new VBC-binding ligands. FP competitive binding experiments were performed on an Infinite F200 Pro instrument (Tecan, Männedorf, Switzerland) in 384-well plates (Corning 3575, Corning, NY, USA), with 485-nm and 520-nm excitation and emission wavelengths (λ), respectively. Each well (20 µL) contained 0.5 µM of VBC protein, 20 nM of FITC-LEALA-HyP-YIPA peptide in 10 mM HEPES at pH 7.5, 0.0025% Tween 20, 50 mM NaCl, 1 mM EDTA, and 1 mM DTT. The control wells contained VBC and peptide in the absence of compound (maximum signal) and peptide in the absence of protein (background signal); VH032 was used as a reference molecule.

A thermal shift assay was conducted to screen the fragment hits selected from the FP assay. The reaction mixture contained 10 µM VBC protein and 500 µM chemicals in reaction buffer (20 mM Bis-Tris pH 7.0, 150 mM NaCl, and 1 mM DTT). In addition, protein thermal shift dye (Protein Thermal Shift™ Dye Kit, 1× final concentration; Applied Biosystem, Waltham, MA, USA) was added to each reaction mixture. The melting temperatures (T_m_) of the proteins were measured using a Quantstudio 6 Real-Time PCR Machine (Applied Biosystems, Waltham, MA, USA).

### X-ray data collection and structure determination

VBC protein crystallization was performed at 14 ℃ using the hanging drop vapor diffusion method. Briefly, each hanging drop was prepared by mixing 1 µL of the protein solution (15–20 mg/mL protein concentration in protein storage buffer) and 1 µL of the reservoir solution (12% PEG 8,000, 100 mM sodium cacodylate, pH 6.0, 200 mM magnesium acetate, and 5 mM DTT). Crystals were observed and grown within 3 days at 14 ℃.

VBC crystals were flash-frozen using a cryoprotectant solution from a crystallization reservoir solution supplemented with 25% (v/v) glycerol. Diffraction data were collected from synchrotron facilities using an Eiger 9 M detector at the Pohang Light Source (Korea) PLS-5 C experimental station and an Eiger X 16 M detector at the Photon Factory (Japan) PF-17 A experimental station. The raw data were processed and scaled using the HKL2000 program suite or XDS^40,41^. The phase was calculated by molecular replacement with the program PHASER^42^using a known VBC structure (PDB entry, 1VCB) as a search model^19^. To determine VBC in complex with chemicals, the VBC crystals were soaked with chemicals (final concentration: 5 mM) for 1 h at 14 ℃ and were subsequently flash-frozen. Co-crystallization was performed to determine the structure of the VBC ligand complex. Further model building was completed using the COOT program^43^and refinement was conducted using phenix. refine in the PHENIX program suite^44^. The coordinates and cif restraint files of the ligands were generated using the eLBOW in the PHENIX program suite^45,46^.

### Surface plasmon resonance experiment

Protein–ligand interactions were investigated using surface plasmon resonance (SPR; SR7500, Reichert, Buffalo, NY, USA). The VBC protein was flowed over the chip surface in an immobilization buffer (10 mM sodium acetate, pH 5.0). This protein was immobilized by standard amine coupling onto a Carboxymethyl Dextran sensor chip (SR7000 GOLD SENSOR SLIDE; Reichert, Buffalo, NY, USA) until saturation (immobilization level ~ 8,000 RU). Before SPR analysis, the chips were equilibrated with running buffer (DMSO 2.5% in 1× PBS). For association analyses, several concentrations of ligand-containing analyte (9.77 µM to 1.25 mM) were flowed over the VBC immobilized chips at 30 µL/min for 2 min. Subsequently, for molecular dissociation analyses, the running buffer was flowed over the chip for an additional 30 µL/min for 3 min. SPR data were analyzed using SCRUBBER2 software (BioNavis, Tampere, Finland).

## Electronic supplementary material

Below is the link to the electronic supplementary material.

Supplementary Material 1

## Data Availability

Coordinates and structural factors were deposited in the RCSB Protein Data Bank under the accession codes 8ZVJ (VBC-7HC_1(DE22) complex), 8ZV8 (VBC-7HC_2(D7) complex), and 9IPW (VBC-7HC_5(D3) complex).

## References

[1] Komander, D. & Rape, M. The ubiquitin code. *Annu. Rev. Biochem.* **81**, 203–229. 10.1146/annurev-biochem-060310-170328 (2012). 22524316 10.1146/annurev-biochem-060310-170328

[2] Popovic, D., Vucic, D. & Dikic, I. Ubiquitination in disease pathogenesis and treatment. *Nat. Med.* **20**, 1242–1253. 10.1038/nm.3739 (2014). 25375928 10.1038/nm.3739

[3] Ciechanover, A. & Ben-Saadon, R. N-terminal ubiquitination: more protein substrates join in. *Trends Cell. Biol.* **14**, 103–106. 10.1016/j.tcb.2004.01.004 (2004). 15055197 10.1016/j.tcb.2004.01.004

[4] Wertz, I. E. & Wang, X. From Discovery to Bedside: targeting the Ubiquitin System. *Cell. Chem. Biol.* **26**, 156–177. 10.1016/j.chembiol.2018.10.022 (2019). 30554913 10.1016/j.chembiol.2018.10.022

[5] Dale, B. et al. Advancing targeted protein degradation for cancer therapy. *Nat. Rev. Cancer*. **21**, 638–654. 10.1038/s41568-021-00365-x (2021). 34131295 10.1038/s41568-021-00365-x PMC8463487

[6] Michaelides, I. N. & Collie, G. W. E3 ligases Meet their Match: fragment-based approaches to Discover New E3 ligands and to unravel E3 Biology. *J. Med. Chem.* **66**, 3173–3194. 10.1021/acs.jmedchem.2c01882 (2023). 36821822 10.1021/acs.jmedchem.2c01882 PMC10009759

[7] Wang, Y., Jiang, X., Feng, F., Liu, W. & Sun, H. Degradation of proteins by PROTACs and other strategies. *Acta Pharm. Sin B*. **10**, 207–238. 10.1016/j.apsb.2019.08.001 (2020). 32082969 10.1016/j.apsb.2019.08.001 PMC7016280

[8] Troup, R. I., Fallan, C. & Baud, M. G. J. Current strategies for the design of PROTAC linkers: a critical review. *Explor. Target. Antitumor Ther.* **1**, 273–312. 10.37349/etat.2020.00018 (2020). 36046485 10.37349/etat.2020.00018 PMC9400730

[9] Bekes, M., Langley, D. R. & Crews, C. M. PROTAC targeted protein degraders: the past is prologue. *Nat. Rev. Drug Discov*. **21**, 181–200. 10.1038/s41573-021-00371-6 (2022). 35042991 10.1038/s41573-021-00371-6 PMC8765495

[10] He, Y. et al. Proteolysis targeting chimeras (PROTACs) are emerging therapeutics for hematologic malignancies. *J. Hematol. Oncol.* **13**, 103. 10.1186/s13045-020-00924-z (2020). 32718354 10.1186/s13045-020-00924-z PMC7384229

[11] Neklesa, T. K., Winkler, J. D. & Crews, C. M. Targeted protein degradation by PROTACs. *Pharmacol. Ther.* **174**, 138–144. 10.1016/j.pharmthera.2017.02.027 (2017). 28223226 10.1016/j.pharmthera.2017.02.027

[12] Buckley, D. L. et al. Small-molecule inhibitors of the interaction between the E3 ligase VHL and HIF1alpha. *Angew Chem. Int. Ed. Engl.* **51**, 11463–11467. 10.1002/anie.201206231 (2012). 23065727 10.1002/anie.201206231 PMC3519281

[13] Buckley, D. L. et al. Targeting the Von Hippel-Lindau E3 ubiquitin ligase using small molecules to disrupt the VHL/HIF-1alpha interaction. *J. Am. Chem. Soc.* **134**, 4465–4468. 10.1021/ja209924v (2012). 22369643 10.1021/ja209924v PMC3448299

[14] Ishida, T. & Ciulli, A. E3 ligase ligands for PROTACs: how they were found and how to Discover New ones. *SLAS Discov*. **26**, 484–502. 10.1177/2472555220965528 (2021). 33143537 10.1177/2472555220965528 PMC8013866

[15] Van Molle, I. et al. Dissecting fragment-based lead discovery at the Von Hippel-Lindau protein:hypoxia inducible factor 1alpha protein-protein interface. *Chem. Biol.* **19**, 1300–1312. 10.1016/j.chembiol.2012.08.015 (2012). 23102223 10.1016/j.chembiol.2012.08.015 PMC3551621

[16] Diehl, C. J. & Ciulli, A. Discovery of small molecule ligands for the Von Hippel-Lindau (VHL) E3 ligase and their use as inhibitors and PROTAC degraders. *Chem. Soc. Rev.* **51**, 8216–8257. 10.1039/d2cs00387b (2022). 35983982 10.1039/d2cs00387b PMC9528729

[17] Lucas, X., Van Molle, I. & Ciulli, A. Surface probing by fragment-based screening and computational methods identifies ligandable pockets on the Von Hippel-Lindau (VHL) E3 ubiquitin ligase. *J. Med. Chem.* **61**, 7387–7393. 10.1021/acs.jmedchem.8b00842 (2018). 30040896 10.1021/acs.jmedchem.8b00842 PMC6109845

[18] Cardote, T. A. F. & Ciulli, A. Structure-guided design of peptides as tools to probe the protein-protein Interaction between Cullin-2 and elongin BC substrate adaptor in Cullin RING E3 Ubiquitin Ligases. *ChemMedChem* **12**, 1491–1496. 10.1002/cmdc.201700359 (2017). 28776949 10.1002/cmdc.201700359 PMC5639367

[19] Stebbins, C. E., Kaelin, W. G. Jr. & Pavletich, N. P. Structure of the VHL-ElonginC-ElonginB complex: implications for VHL tumor suppressor function. *Science* **284**, 455–461. 10.1126/science.284.5413.455 (1999). 10205047 10.1126/science.284.5413.455

[20] Adasme, M. F. et al. PLIP 2021: expanding the scope of the protein-ligand interaction profiler to DNA and RNA. *Nucleic Acids Res.* **49**, W530–W534. 10.1093/nar/gkab294 (2021). 33950214 10.1093/nar/gkab294 PMC8262720

[21] Vogt, A. D. & Di Cera, E. Conformational selection or induced fit? A critical appraisal of the kinetic mechanism. *Biochemistry* **51**, 5894–5902. 10.1021/bi3006913 (2012). 22775458 10.1021/bi3006913 PMC3550001

[22] Takagi, Y., Conaway, J. W. & Conaway, R. C. A novel activity associated with RNA polymerase II elongation factor SIII. SIII directs promoter-independent transcription initiation by RNA polymerase II in the absence of initiation factors. *J. Biol. Chem.* **270**, 24300–24305. 10.1074/jbc.270.41.24300 (1995). 7592640 10.1074/jbc.270.41.24300

[23] Garrett, K. P., Haque, D., Conaway, R. C. & Conaway, J. W. A human cDNA encoding the small subunit of RNA polymerase II transcription factor SIII. *Gene* **150**, 413–414. 10.1016/0378-1119(94)90467-7 (1994). 7821821 10.1016/0378-1119(94)90467-7

[24] Reines, D., Conaway, J. W. & Conaway, R. C. The RNA polymerase II general elongation factors. *Trends Biochem. Sci.* **21**, 351–355 (1996). 8870500 PMC3374595

[25] Chen, Y. et al. Structure of the transcribing RNA polymerase II-Elongin complex. *Nat. Struct. Mol. Biol.* **30**, 1925–1935. 10.1038/s41594-023-01138-w (2023). 37932450 10.1038/s41594-023-01138-w PMC10716050

[26] Jang, S. M., Redon, C. E. & Aladjem, M. I. Chromatin-bound Cullin-Ring ligases: Regulatory roles in DNA replication and potential targeting for Cancer Therapy. *Front. Mol. Biosci.* **5**, 19. 10.3389/fmolb.2018.00019 (2018). 29594129 10.3389/fmolb.2018.00019 PMC5859106

[27] Cai, W. & Yang, H. The structure and regulation of Cullin 2 based E3 ubiquitin ligases and their biological functions. *Cell. Div.* **11**, 7. 10.1186/s13008-016-0020-7 (2016). 27222660 10.1186/s13008-016-0020-7 PMC4878042

[28] Mahrour, N. et al. Characterization of Cullin-box sequences that direct recruitment of Cul2-Rbx1 and Cul5-Rbx2 modules to Elongin BC-based ubiquitin ligases. *J. Biol. Chem.* **283**, 8005–8013. 10.1074/jbc.M706987200 (2008). 18187417 10.1074/jbc.M706987200

[29] Nguyen, H. C., Yang, H., Fribourgh, J. L., Wolfe, L. S. & Xiong, Y. Insights into Cullin-RING E3 ubiquitin ligase recruitment: structure of the VHL-EloBC-Cul2 complex. *Structure* **23**, 441–449. 10.1016/j.str.2014.12.014 (2015). 25661653 10.1016/j.str.2014.12.014 PMC4351159

[30] Kim, Y. K. et al. Structural basis of intersubunit recognition in elongin BC-cullin 5-SOCS box ubiquitin-protein ligase complexes. *Acta Crystallogr. D Biol. Crystallogr.* **69**, 1587–1597. 10.1107/S0907444913011220 (2013). 23897481 10.1107/S0907444913011220

[31] Cardote, T. A. F., Gadd, M. S. & Ciulli, A. Crystal Structure of the Cul2-Rbx1-EloBC-VHL Ubiquitin Ligase Complex. *Structure* 25, 901–911 e903, (2017). 10.1016/j.str.2017.04.009 10.1016/j.str.2017.04.009 PMC5462531 28591624

[32] Kornicka, A., Balewski, L., Lahutta, M. & Kokoszka, J. Umbelliferone and its synthetic derivatives as suitable molecules for the Development of agents with Biological activities: a review of their pharmacological and therapeutic potential. *Pharmaceuticals (Basel)*. 16. 10.3390/ph16121732 (2023). 10.3390/ph16121732 PMC10747342 38139858

[33] Ghani, U. et al. Crystal structure of gamma-chymotrypsin in complex with 7-hydroxycoumarin. *J. Mol. Biol.* **314**, 519–525. 10.1006/jmbi.2001.5148 (2001). 11846564 10.1006/jmbi.2001.5148

[34] Patterson, E. I. et al. Structural characterization of beta-ketoacyl ACP synthase I bound to platencin and fragment screening molecules at two substrate binding sites. *Proteins* **88**, 47–56. 10.1002/prot.25765 (2020). 31237717 10.1002/prot.25765 PMC9518911

[35] Berger, I., Guttman, C., Amar, D., Zarivach, R. & Aharoni, A. The molecular basis for the broad substrate specificity of human sulfotransferase 1A1. *PLoS One*. **6**, e26794. 10.1371/journal.pone.0026794 (2011). 22069470 10.1371/journal.pone.0026794 PMC3206062

[36] Bulatov, E., Ciulli, A. & Targeting Cullin -RING E3 ubiquitin ligases for drug discovery: structure, assembly and small-molecule modulation. *Biochem. J.* **467**, 365–386. 10.1042/BJ20141450 (2015). 25886174 10.1042/BJ20141450 PMC4403949

[37] Soucy, T. A. et al. An inhibitor of NEDD8-activating enzyme as a new approach to treat cancer. *Nature* **458**, 732–736. 10.1038/nature07884 (2009). 19360080 10.1038/nature07884

[38] Xiao, Z. et al. 7-Hydroxycoumarins are Affinity-based fluorescent probes for competitive binding studies of Macrophage Migration Inhibitory factor. *J. Med. Chem.* **63**, 11920–11933. 10.1021/acs.jmedchem.0c01160 (2020). 32940040 10.1021/acs.jmedchem.0c01160 PMC7586407

[39] Li, J. et al. Cullin-RING ligases employ geometrically optimized catalytic partners for substrate targeting. *Mol Cell* 84, 1304–1320 e1316, (2024). 10.1016/j.molcel.2024.01.022 10.1016/j.molcel.2024.01.022 PMC10997478 38382526

[40] Otwinowski, Z. & Minor, W. Processing of X-ray diffraction data collected in oscillation mode. *Methods Enzymol.* **276**, 307–326. 10.1016/S0076-6879(97)76066-X (1997). 27754618 10.1016/S0076-6879(97)76066-X

[41] Kabsch, W. & Xds *Acta Crystallogr. D Biol. Crystallogr.* **66**, 125–132, doi:10.1107/S0907444909047337 (2010). 20124692 10.1107/S0907444909047337 PMC2815665

[42] McCoy, A. J. et al. Phaser crystallographic software. *J. Appl. Crystallogr.* **40**, 658–674. 10.1107/S0021889807021206 (2007). 19461840 10.1107/S0021889807021206 PMC2483472

[43] Emsley, P., Lohkamp, B., Scott, W. G. & Cowtan, K. Features and development of Coot. *Acta Crystallogr. D Biol. Crystallogr.* **66**, 486–501. 10.1107/S0907444910007493 (2010). 20383002 10.1107/S0907444910007493 PMC2852313

[44] Afonine, P. V. et al. Towards automated crystallographic structure refinement with phenix.refine. *Acta Crystallogr. D Biol. Crystallogr.* **68**, 352–367. 10.1107/S0907444912001308 (2012). 22505256 10.1107/S0907444912001308 PMC3322595

[45] Adams, P. D. et al. PHENIX: a comprehensive Python-based system for macromolecular structure solution. *Acta Crystallogr. D Biol. Crystallogr.* **66**, 213–221. 10.1107/S0907444909052925 (2010). 20124702 10.1107/S0907444909052925 PMC2815670

[46] Moriarty, N. W., Grosse-Kunstleve, R. W. & Adams, P. D. Electronic Ligand Builder and Optimization Workbench (eLBOW): a tool for ligand coordinate and restraint generation. *Acta Crystallogr. D Biol. Crystallogr.* **65**, 1074–1080. 10.1107/S0907444909029436 (2009). 19770504 10.1107/S0907444909029436 PMC2748967

