# Author Correction: Identification of novel 7-hydroxycoumarin derivatives as ELOC binders with potential to modulate CRL2 complex formation

**DOI:** 10.1038/s41598-025-92597-2

**Published:** 2025-03-07

**Authors:** Yonghyeok Kim, Seon Jeong Baek, Eun-Kyung Yoon, Minhee Choi, Jung-Hoon Kim, Kyungtae Kim, Chi Hoon Park, Byung Il Lee

**Affiliations:** 1 Research Institute, National Cancer Center, Goyang-Si, 10408 Gyeonggi Republic of Korea; 2 Department of Cancer Biomedical Science, National Cancer Center Graduate School of Cancer Science and Policy, Goyang-Si, 10408 Gyeonggi Republic of Korea; 3 Department of Medicinal Chemistry and Pharmacology, University of Science and Technology, Daejeon, 34113 Republic of Korea; 4 Therapeutics & Biotechnology Division, Korea Research Institute of Chemical Technology, Daejeon, 34114 Republic of Korea

Correction to: *Scientific Reports* 10.1038/s41598-025-88166-2, published online 29 January 2025

The original version of this Article contained errors in the Figures. Figure 1 was published as Figure 2 and Figure 2 was published as Figure 1. The Figure legends were correct at the time of publication.

The original Figures 1 and 2 and accompanying legends appear below.

**Fig. 1:**
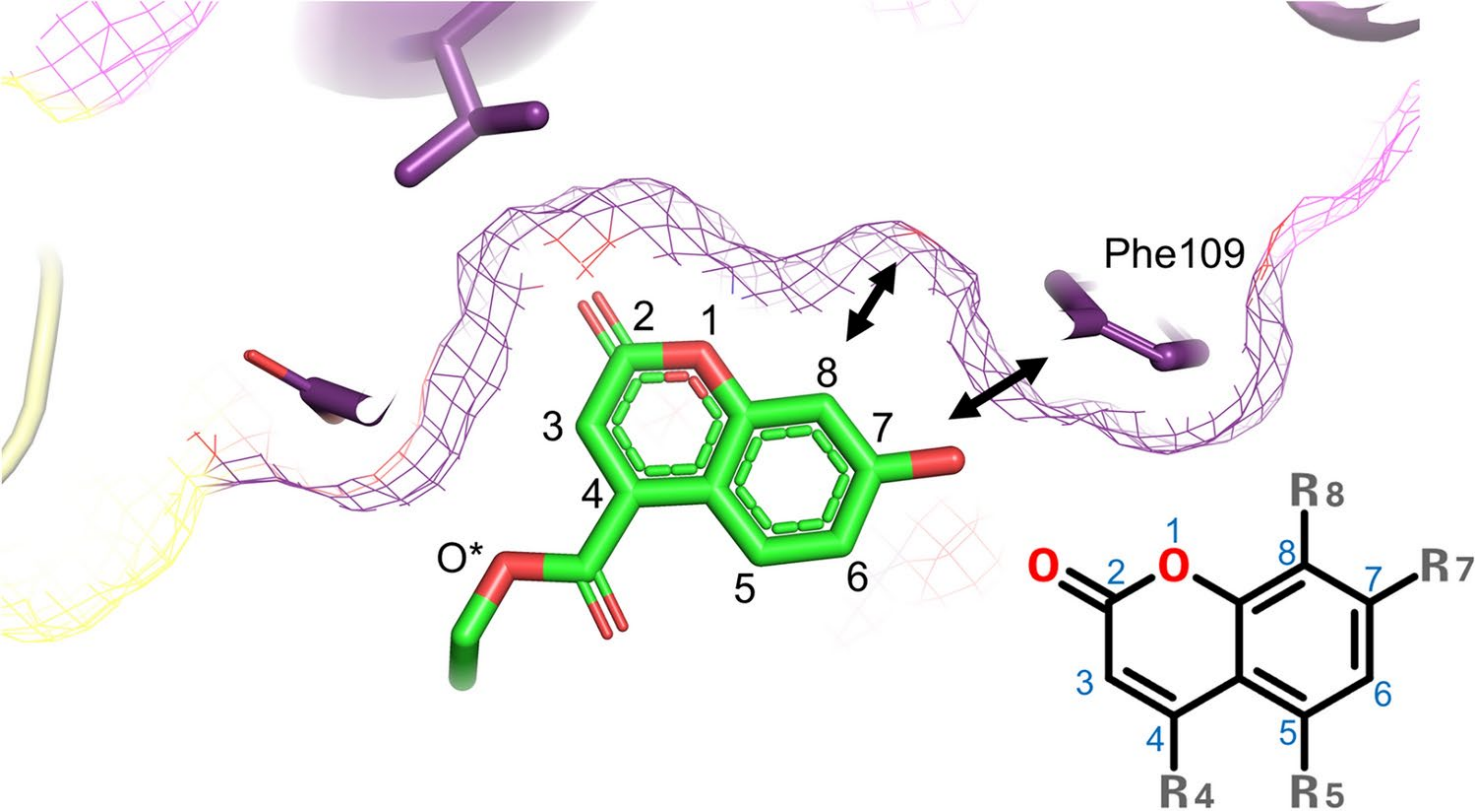
Overall structures of VBC in complex with fragment ligands. (**a**) Overall structures of the VBC−7HC_1(DE22) complex. Each protein component is colored in cyan (VHL), pale yellow (ELOB), and violet (ELOC). The ligand is shown in the sphere model. (**b**) Chemical structures and 2mFo-DFc OMIT maps for 7HC_1(DE22), 7HC_2(D7), and 7HC_5(D3). The numbering for the 7HC ring is also shown. The map contour levels were 1.5 σ. (**c**) Superimposition with ligand-bound VBC structures. 7HC_1(DE22) is drawn in green, 7HC_2(D7) in yellow, 7HC_5(D3) in cyan, and MB235 in black (PDB code: 6GMN)^17^. O*: position of the O3 atom in the 7HC_1(DE22) and 7HC_1(DE22) molecules and O4 atom in the 7HC_5(D3) molecule. (**d**) Detailed views of interactions between VBC and 7HC_1(DE22). (**e**) Detailed views of interactions between VBC and 7HC_2(D7).

**Fig. 2:**
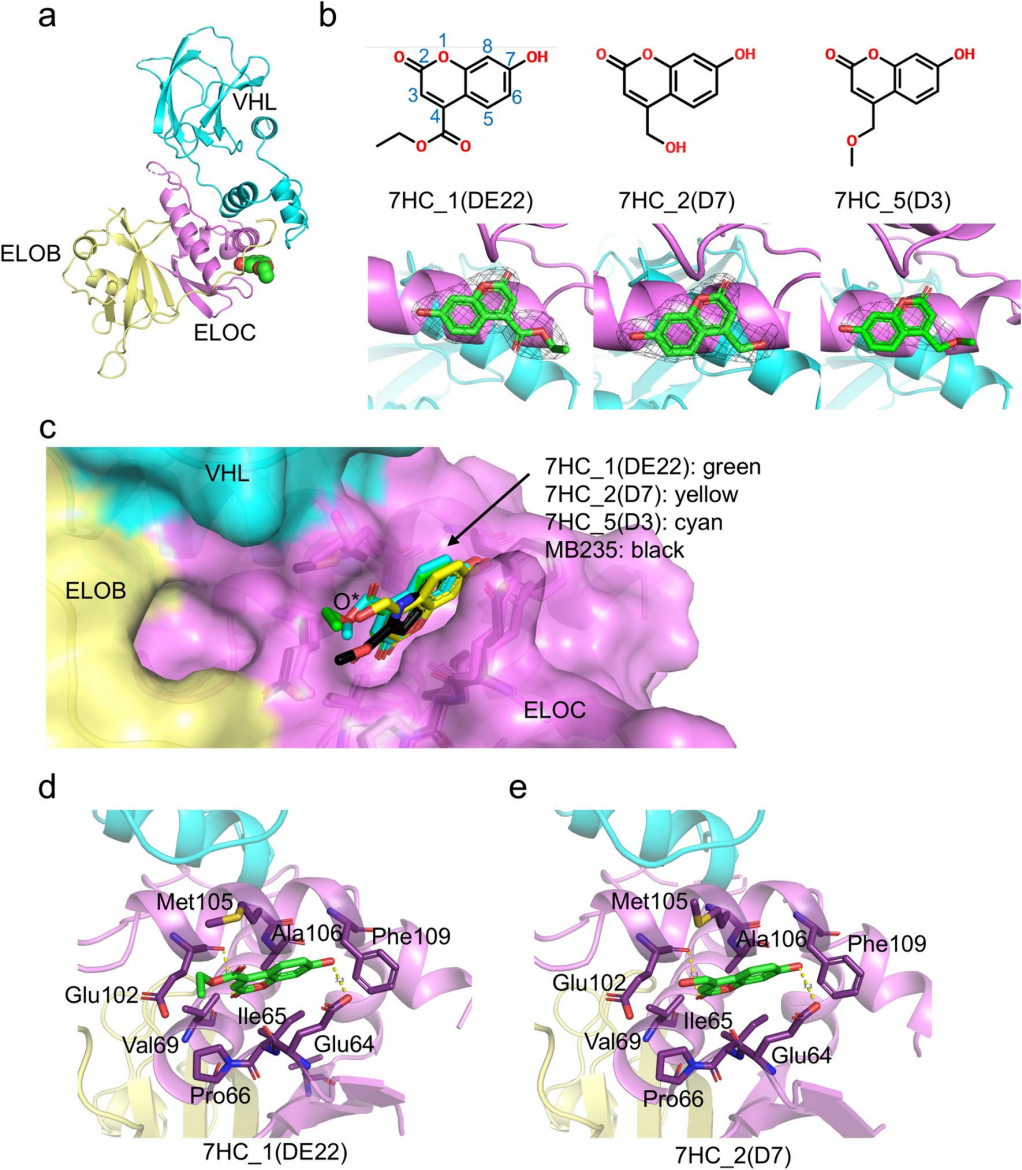
Sliced view of 7HC derivative binding pocket surface showing the structural basis of no binding activities by C7- and C8-position chemical modifications (R7 and R8). The 7HC_1(DE22) structure is shown as the representative. O*: position of the O3 atom in the 7HC_1(DE22).

The original Article has been corrected.

