# Supplementary material for: Identification of novel 7-hydroxycoumarin derivatives as ELOC binders with potential to modulate CRL2 complex formation
Source: Sci Rep. 2025 Jan 29;15:3622. doi: 10.1038/s41598-025-88166-2 (PMC11779939; doi:10.1038/s41598-025-88166-2)
Supplement: Supplementary file 1 — Supplementary Material 1 [file 41598_2025_88166_MOESM1_ESM.docx]

**Table S1. List of 7HC derivatives in this study**

| **Compound name** | **IUPAC name/**  **InChlKey** | **Chemical structure** | **Studies attempted** |
| --- | --- | --- | --- |
| 7HC_1(DE22) | ethyl 7-hydroxy-2-oxo-2H-chromene-4-carboxylate/  SNMUERBNQDOIQB-UHFFFAOYSA-N | 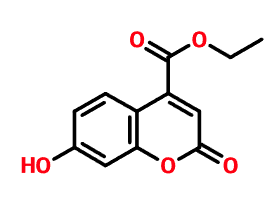 | X-ray (ligand density map)  SPR (weak binding, Not determined K_D_) |
| 7HC_2(D7) | 7-Hydroxy-4-(hydroxymethyl)-2H-chromen-2-one/  IWPNSXBCZANXBC-UHFFFAOYSA-N | 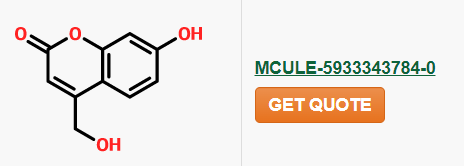 | X-ray (ligand density map)  SPR (weak binding, Not determined K_D_) |
| 7HC_3(D1) | 4-(Chloromethyl)-7-hydroxy-2H-chromen-2-one/  TXSLBPGPBNGHRW-UHFFFAOYSA-N | 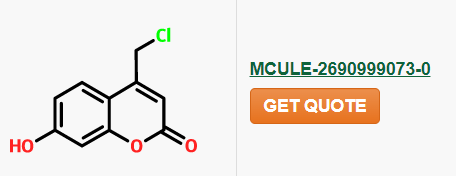 | X-ray (no density map) |
| C_4(D2) | 2-((4-ethyl-2-oxo-2H-chromen-7-yl)oxy)propanoic acid/  XXYKRZPDNGYPRH-UHFFFAOYSA-N | 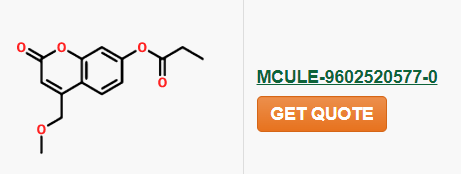 | X-ray (no density map) |
| 7HC_5(D3) | 7-hydroxy-4-(methoxymethyl)chromen-2-one/  PRVOZGKAZHXKMM-UHFFFAOYSA-N | 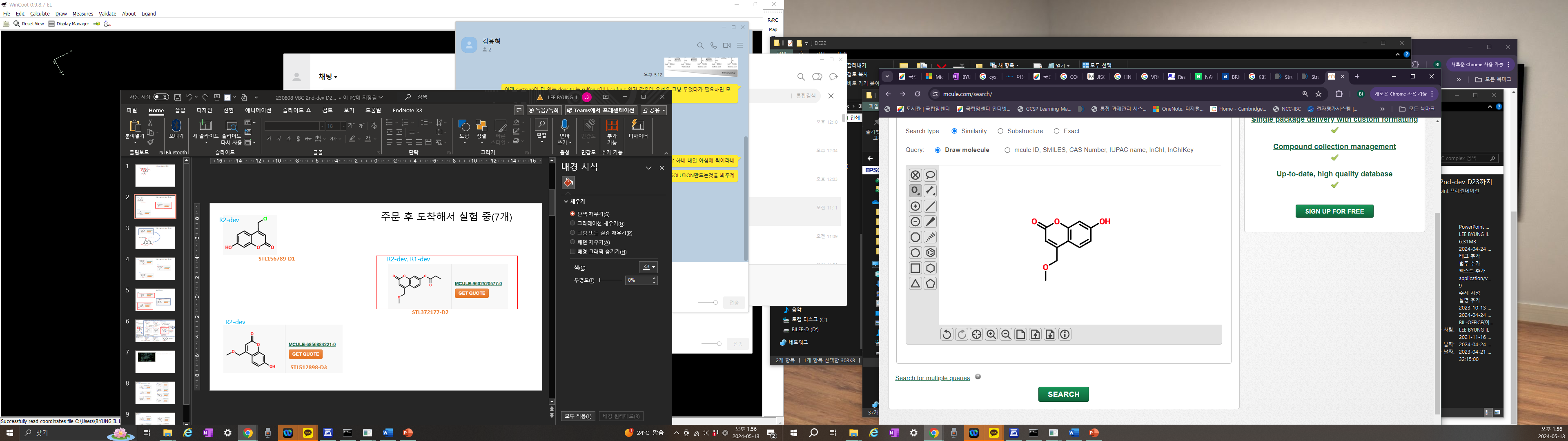 | X-ray (ligand density map)  SPR (weak & non-specific binding signal, Not determined K_D_) |
| 7HC_6(D4) | 5,7-Dihydroxy-4-methyl-2H-chromen-2-one/  QNVWGEJMXOQQPM-UHFFFAOYSA-N | 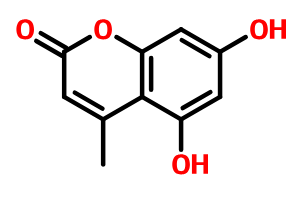 | X-ray (no density map)  SPR (no binding) |
| 7HC_7(D5) | methyl 2-(7-hydroxy-2-oxochromen-4-yl)acetate/  YRNMDWOVAZLMDY-UHFFFAOYSA-N | 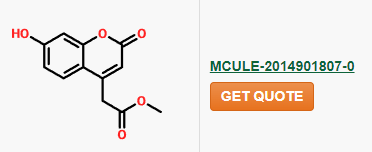 | X-ray (no density map)  SPR (no binding) |
| 7HC_8(D6) | 4-Ethyl-7-hydroxycoumarin/  UNRDBISCGQHNDA-UHFFFAOYSA-N | 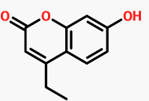 | X-ray (no density map) |
| C_9(D8) | ethyl 7-amino-2-oxo-2H-chromene-4-carboxylate/  KYCTVTQDTGLRNT-UHFFFAOYSA-N | 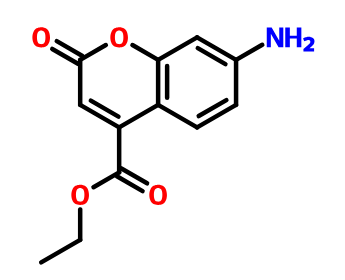 | X-ray (no density map)  SPR (no binding) |
| C_10(D9) | Ethyl 7-azido-2-oxo-2H-chromene-4-carboxylate/  AQYHYGULAUAPPO-UHFFFAOYSA-N | 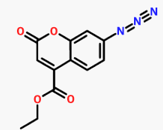 | X-ray (no density map) |
| C_11(D10) | 8-Hydroxy-2-oxo-2 h-chromene-4-carboxylic acid/  NCLALGFNMYEPBH-UHFFFAOYSA-N | 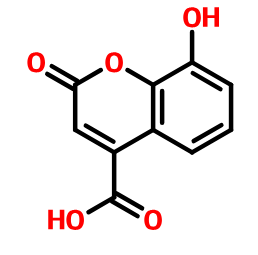 | X-ray (no density map) |
| C_12(D11) | 7-Amino-4-(methoxymethyl)-2H-chromen-2-one/  QZZLLHOMMCKWIQ-UHFFFAOYSA-N | 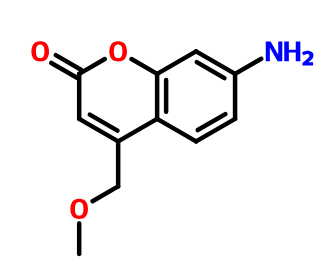 | X-ray (no density map) |
| C_13(D12) | 4-methyl-7-(methylamino)-2H-chromen-2-one/  LKJDPYZBJFUZNS-UHFFFAOYSA-N | 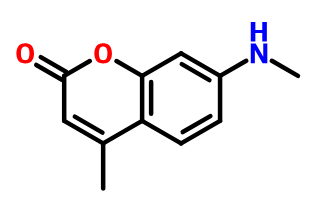 | X-ray (no density map) |
| C_14(D13) | 7-isocyanato-4-methyl-2H-chromen-2-one/  YCEHPUBNERNXNP-UHFFFAOYSA-N | 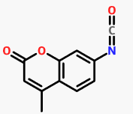 | X-ray (no density map) |
| C_15(D14) | 7-(Diethylamino)-4-(hydroxymethyl)-2H-chromen-2-one/  NMZSXNOCNJMJQT-UHFFFAOYSA-N | 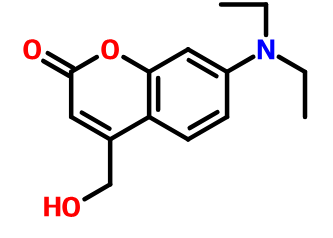 | X-ray (no density map) |
| C_16(D15) | 7-(ethylamino)-4-methyl-2H-chromen-2-one/  OTNIKUTWXUODJZ-UHFFFAOYSA-N | 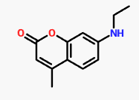 | X-ray (no density map) |
| C_17(D16) | 7-isothiocyanato-4-methyl-2H-chromen-2-one/  RZODWTHSAMFLEZ-UHFFFAOYSA-N | 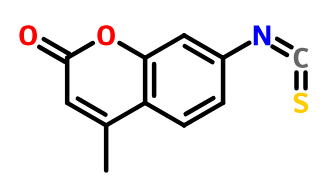 | X-ray (no density map) |
| C_18(D17) | 7-(Dimethylamino)-4-methylcoumarin/  GZEYLLPOQRZUDF-UHFFFAOYSA-N | 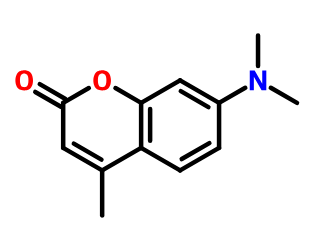 | X-ray (no density map) |
| C_19(D18) | 7-Methoxy-4,8-dimethylchromen-2-one/  OMNGMJMBWPLZLA-UHFFFAOYSA-N | 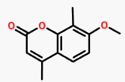 | X-ray (no density map) |
| 7HC_20(D19) | 7-Hydroxy-4,8-dimethyl-2H-chromen-2-one/  MVMMGVPSTRNMSV-UHFFFAOYSA-N | 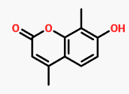 | X-ray (no density map) |
| 7HC_21(D20) | 4-(chloromethyl)-7-hydroxy-8-methyl-2H-chromen-2-one/  JVVZWJIRFMWXCT-UHFFFAOYSA-N | 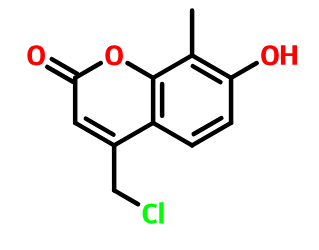 | X-ray (no density map)  SPR (no binding) |
| 7HC_22(D21) | 4-butyl-7-hydroxy-8-methyl-2H-chromen-2-one/  MDWSJIOFAJKBJY-UHFFFAOYSA-N | 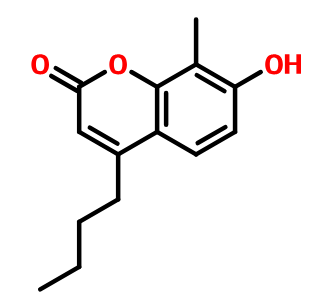 | X-ray (no density map) |
| 7HC_23(D22) | 8-Acetyl-7-hydroxy-4-methyl-2H-chromen-2-one/  WZOMQVFUPMLOGT-UHFFFAOYSA-N | 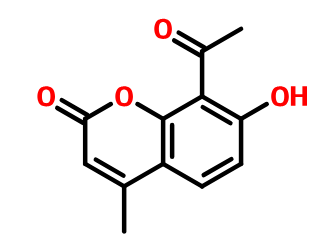 | X-ray (no density map)  SPR (no binding) |
| 7HC_24(D23) | 2-(7-hydroxy-8-methyl-2-oxo-2H-chromen-4-yl)acetamide/  WJSDERGBGZQYPS-UHFFFAOYSA-N | 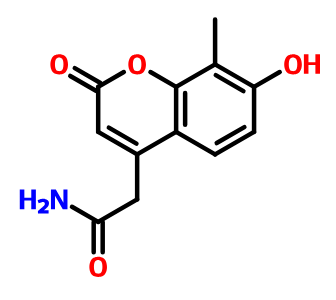 | X-ray (no density map) |
| 7HC_25(org); hit compound from FBDD library | 7-hydroxy-4-(trifluoromethyl)chromen-2-one/  CCKWMCUOHJAVOL-UHFFFAOYSA-N | 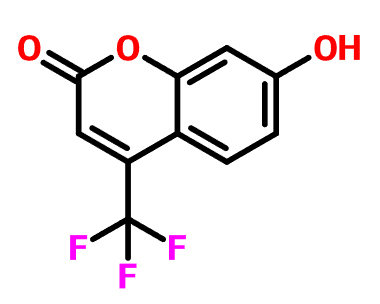 | X-ray (no density map) |

**Table S2. Data collection and refinement statistics**

| **Dataset** | **7HC_1(DE22)** | **7HC_2(D7)** | **7HC_5(D3)** |
| --- | --- | --- | --- |
| **Diffraction data statistics** |  |  |  |
| X-ray source | PLS-BL5C | PLS-BL5C | PF-BL17A |
| Wavelength (Å) | 1.00003 | 1.00003 | 0.98000 |
| Space group | P4_1_22 | P4_1_22 | P4_1_22 |
| Cell parameters |  |  |  |
| a, b, c (Å) | 93.733, 93.733, 364.159 | 93.896, 93.896, 364.118 | 94.443, 94.443, 361.486 |
| α, β, γ (°) | 90, 90, 90 | 90, 90, 90 | 90, 90, 90 |
| Resolution range (Å) | 50.0–2.60 (2.64–2.60)* | 50.0–2.46 (2.50–2.46)* | 50.0–3.00 (3.00–3.18)* |
| R_sym_ (%) | 12.8 (81.5) | 9.2 (55.5) | 16.7 (70.6) |
| CC_1/2_ | 1.000 (0.894) | 0.996 (0.860) | 0.998 (0.782) |
| Mean I/σI | 15.429 (2.500) | 18.736 (2.818) | 9.46 (2.21) |
| Redundancy | 7.1 (7.8) | 7.9 (7.7) | 5.8 (5.1) |
| Completeness (%) | 99.8 (100.0) | 99.6 (100.0) | 99.8 (99.2) |
| No. of total/unique reflections | 364,340/51,235 | 475,483/60,319 | 364,144/62,450 |
| **Refinement statistics** |  |  |  |
| Resolution range (Å) | 38.08–2.60 | 37.54–2.46 | 49.06–3.00 |
| R_work_/R_free_ (%) | 22.81/28.49 | 23.46/28.64 | 20.70/27.32 |
| No. of nonhydrogen atoms/average B-factor (Å^2^) |  |  |  |
| Protein | 10,399/42.03 | 10,206/39.42 | 10,206/51.88 |
| Water | 57/29.02 | 90/28.34 | 2/51.62 |
| Ligand | 51/40.83 | 56/41.77 | 15/30.85 |
| RMS deviation |  |  |  |
| Bond length (Å) | 0.029 | 0.003 | 0.011 |
| Bond angles (°) | 1.58 | 0.52 | 1.28 |
| Ramachandran plot (%) |  |  |  |
| Favored/outliers | 96.34/0.00 | 96.59/0.00 | 91.82/0.43 |
| Clash score | 3.84 | 4.06 | 11.43 |
| PDB entry | 8ZVJ | 8ZV8 | 9IPW |

*Values in parentheses are for the highest-resolution shell.

**
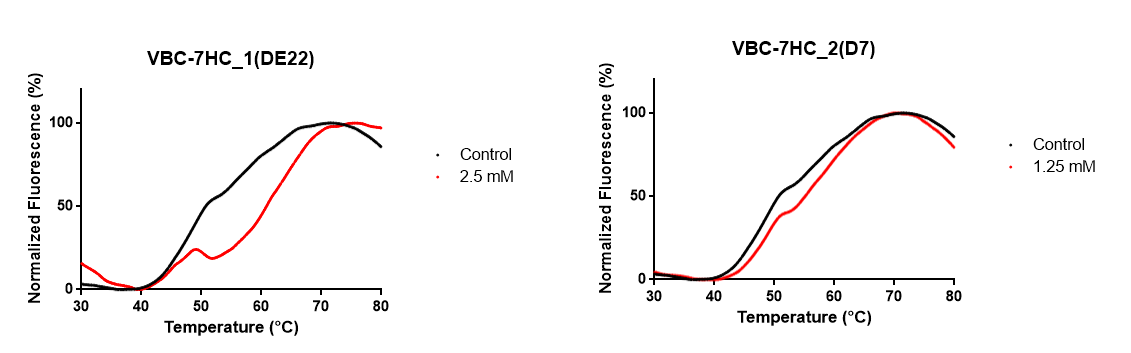
**

**Figure S1.** Representative thermal shift curves illustrating the interactions between VBC−7HC_1(DE22) and VBC−7HC_2(D7).


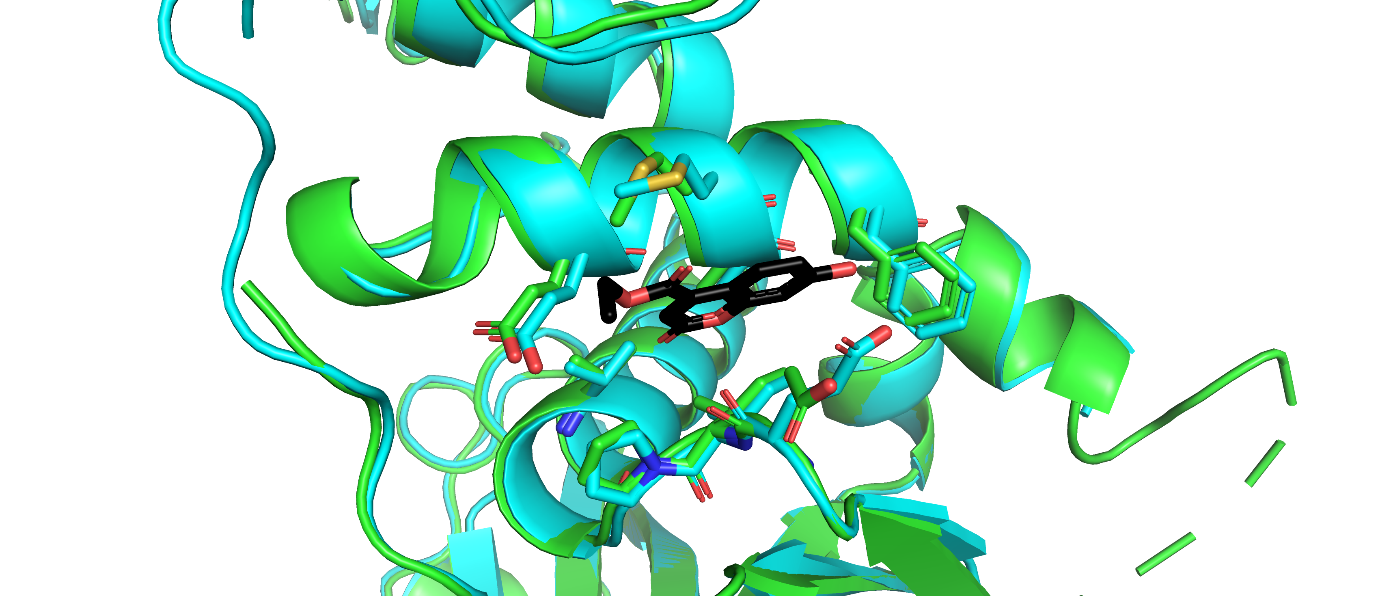


**Figure S2.** Structural superposition of VBC−7HC_1(DE22) (colored in cyan) and ligand-free VBC structures (PDB code, 1VCB, colored in Green).


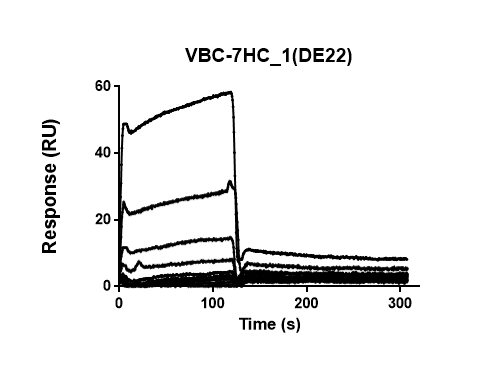

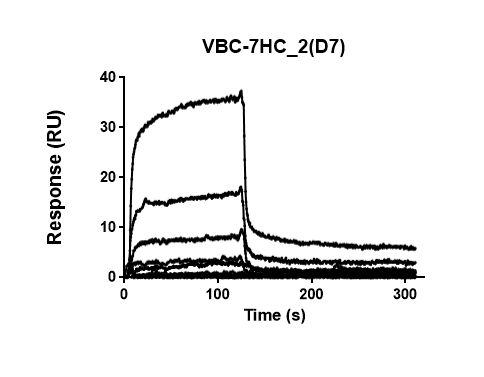


VBC-7HC_1(DE22)

VBC-7HC_2(D7)

Response (RU)

Response (RU)

Time (s)

Time (s)

**Figure S3.** *In vitro* binding activity of 7HCs to ELOC. Monitoring direct protein-ligand interactions by SPR experiments. All SPR experiments were performed in triplicate. K_D_ values could not be calculated because the SPR responses did not reach saturation, which prevented analysis using equilibrium fitting for weak affinities. Additionally, conducting SPR experiments at high ligand concentrations was not feasible due to the poor solubility of the ligands.

**
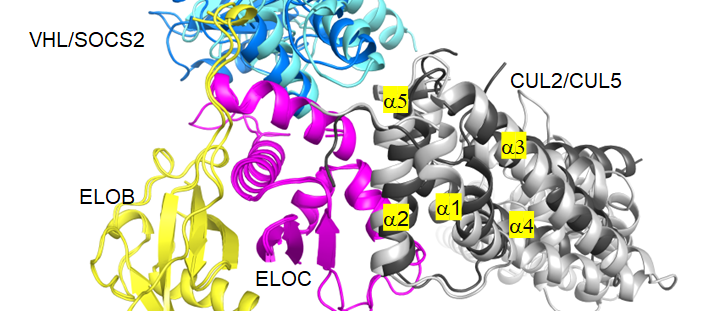
**

**Figure S4.** Structural superposition of VBC−CUL2 and SBC−CUL5 complex structures with the ELOC structure as the central figure (PDB codes, 4WQO and 4JGH). Each component proteins were drawn in cyan (VHL), blue (SOCS2), magenta (ELOC), black (CUL2), and grey (CUL5), respectively. Helices (α1, α2, α4, and α5) in CUL2 and CUL5 structures were indicated.


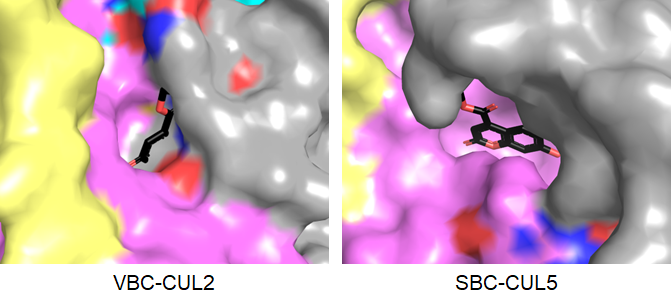


**Figure S5.** The 7HC binding pockets in VBC−CUL2 (PDB code, 4WQO) and SBC−CUL5 (PDB code, 4JGH). Each component proteins were drawn in pale yellow (ELOB), magenta (ELOC), and grey (CUL2 or CUL5), respectively. 7HC_1(DE22) molecule is drawn in black, showing the overlapping with VBC−CUL2.

**
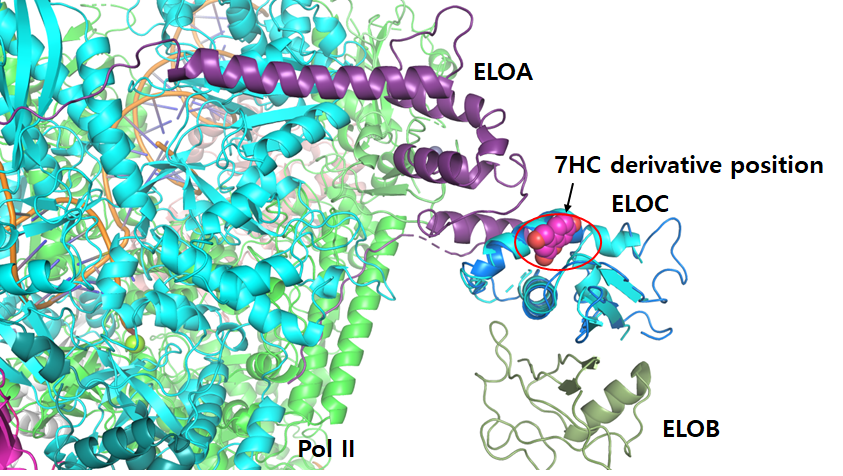
**

**Figure S6.** Structural superposition of mammalian Pol II-SPT6-Elongin complex (PDB code, 8OF0) with 7HC_2(D7) bound ELOC structure.

**
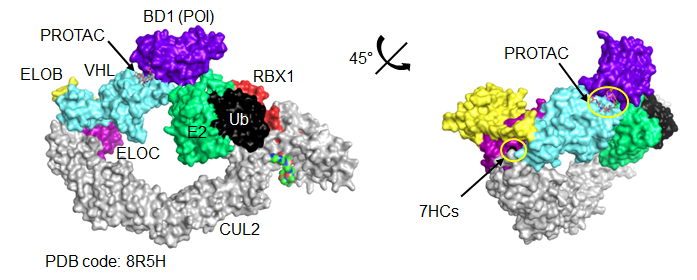
**

**Figure S7.** Structure of CRL2^VHL^ E3 ligase holoenzyme (PDB code, 8R5H). 7HCs binding and PROTAC molecule binding site are indicated.
